# Use of AR/VR for Treatment of Freezing of Gait (FoG) in Parkinson’s Disease (PD)

**DOI:** 10.3390/jcm15052076

**Published:** 2026-03-09

**Authors:** Ayusha Pokharel, Aanya Tamrakar, Nipun Chopra

**Affiliations:** Department of Psychology and Neuroscience, DePauw University, Greencastle, IN 46135, USA; ayushapokharel_2027@depauw.edu (A.P.); aanyatamrakar_2028@depauw.edu (A.T.)

**Keywords:** Freezing of Gait (FoG), Parkinson’s disease, wearable technology, AR/VR

## Abstract

Parkinson’s disease (PD) is the fastest-growing neurodegenerative disease affecting 90 thousand new Americans each year. PD includes motor and non-motor symptoms, resulting in progressive disability and difficulty in completing activities of daily living. Freezing of Gait (FoG) is one of the common disabling symptoms of PD, characterized by difficulties in initiating walking, resulting in gait abnormalities and increased risk of falling (RoF) and fear of falling (FoF). Clinical management of FoG is difficult as it is minimally responsive to both pharmacological and surgical interventions. In fact, these interventions can paradoxically worsen of FoG. Additionally, PD patients with FoG have reported worse health-related quality of life (HR-QoL) due to limitations in mobility, activities of daily living (ADL), bodily discomfort, stigma, and social isolation. Despite its increasing treatment and management of FoG is difficult due to its paroxysmal and heterogeneous nature. Therefore, there is a growing need for effective, evidence-based management and intervention approaches for FoG. Some current techniques used to manage FoG are physical therapy, exercise, gait training, and balance training; however, due to a lack of patient adherence, accessibility concerns, and the need for continuous supervision and individualized feedback, the long-term effectiveness of these interventions remains limited and challenging to achieve in real-world settings. A new promising avenue for managing PD is the use of wearable technology, which can provide audiovisual, via augmented and virtual reality (AR/VR), and tactical cueing to offset FoG, thereby enhancing independence in PD patients. In this comprehensive review, we will provide an overview of the symptoms, monitoring, and treatment of PD, with a focus on the neuroanatomy and treatment of FoG. We will review and critique the extant literature on the use of AR/VR technology in the management of FoG. Finally, the challenges and risks associated with wearable technology in FoG management will also be identified.

## 1. Introduction

**Figure 1:**
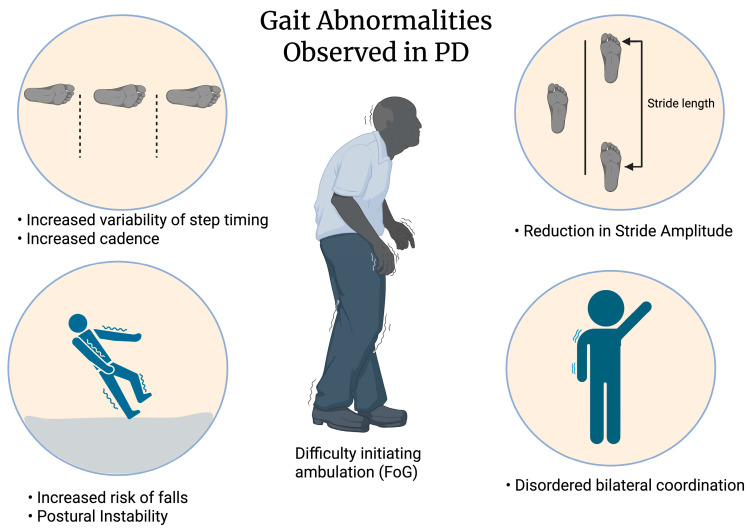
Motor symptoms of Parkinson’s disease include various gait abnormalities. Some types. of gait disturbances observed are increased cadence and variability of step timing, reduced stride amplitude and disordered bilateral coordination. Freezing of gait (FoG) is another motor symptom of PD, which is known to occur in later stages of PD. All these symptoms affect postural stability and increase the risk of falls in PD patients.

PD is a progressive neurodegenerative disorder resulting from the loss of dopaminergic neurons in the substantia nigra due to the aggregation of misfolded *α* -synuclein in Lewy bodies [1,2,3,4]. PD is characterized by motor symptoms (Figure 1; e.g., bradykinesia, tremor, rigidity, and postural instability) and non-motor symptoms (e.g., cognitive changes, mood changes, and autonomic dysfunction) [1,2]. Freezing of Gait (FoG) is defined as “brief, episodic absence or marked reduction of forward progression of the feet despite the intention to walk” [5,6,7]. FoG occurs in patients with PD and other non-PD disorders like progressive supranuclear palsy, multiple system atrophy, ischemic stroke and other neuroinflammatory disease [8]. FoG occurs in up to 63% of idiopathic PD cases [9,10]. Due to the paroxysmal and heterogeneous nature of FoG, the mechanism of FoG is poorly understood; however, both neuronal miscommunication and cognitive overload are hypothesized as potential FoG mechanisms [5,11,12,13]. The risk for fall injuries and injury-related mortalities increases due to FoG [2,10,14]. Additionally, FoG negatively affects both mobility and independence of patients resulting in an overall reduction in the quality of life for PD patients [12].

FoG contributes to worse QoL in PD patients as the severity and frequency of FoG tends to limit activity of daily life, reduce mobility and thereby increase patient’s dependence on others [15,16,17]. Beyond the expected relationship with mobility, bodily discomfort, ADL, FoG severity is associated with (Parkinson’s Disease Questionnaire (PDQ)-39 QoL domains “emotional”, “cognition”, “stigma”, and “communication” [16]. Additionally, PD patients with FoG exhibit more stumbling strides, smaller turning angles and more unpredictability in step timing, all of which raise the risk of instability and falls during daily activities [18]. Therefore, FoG also increases the risk of falling in PD patients and these falls negatively affect patients HR-QoL [17,19,20]. Furthermore, approximately 70% of PD patients have Fear of Falling (FoF) that limits activity and social involvement [21]. At least partly due to FoF, multiple studies have reported that PD patients avoid both community ambulation and activities to minimize the occurrence of FoG and falls, which often leads to social isolation and increases caregiver burden [15,22,23,24]. 

Research on device-aided therapies has shown that external cueing is an effective strategy to reduce FoG induced by PD, as cueing changes habitual walking into goal directed behavior, which helps the brain bypass the damaged basal ganglia and thereby prevents the occurrence of FoG [11,12]. The advancement in the field of Augmented Reality (AR) and Virtual Reality (VR) technologies provides us with a unique opportunity to provide dynamic, versatile cues which are not limited by physical constraints [25]. VR is defined as a “computer-generated simulation of a three-dimensional environment that can be interacted with in a seemingly real or physical way” whereas AR is a technology that overlays digital information onto the real world [26]. In this review, we aim to explore a new potential avenue of rehabilitation via wearable technologies with a major focus on AR/VR.

Following an introduction to PD pathophysiology in Section 2, we explore the current available treatment options and limitations of those therapies in addressing FoG. We briefly review research focusing on rehabilitation to improve the quality of life of PD patients. Section 3 highlights wearable technologies that have been used in PD management. Studying FoG in clinical settings is limited by the lack of objective metrics to measure and induce FoG. Therefore, in Section 4, we examine current approaches used to measure and investigate FoG. In this section, we highlight that AR/VR can be used to induce FoG in laboratory settings to experimentally investigate the effectiveness of FoG mitigation tools. In Section 5, we review and critique the extant literature on the use of AR/VR technology in the management and treatment of FoG. Finally, we highlight the challenges and risks associated with wearable technology in FoG management in Section 6.

## 2. Current Treatment Approaches and Limitations

**Pharmacological:** Levodopa (L-dopa) remains the first choice for treatment of PD [27]. Levodopa’s low bioavailability after oral consumption is proposed as one mechanism that explains its variability in attenuation of PD motor symptoms [2,5,27,28,29]. Consequently, L-dopa was paired with carbidopa, MAO-B inhibitors, and/or COMT inhibitors to increase bioavailability [27].

Despite adverse effects like impulse control disorder and dopamine agonist withdrawal symptoms, dopamine agonists are often used because they produce fewer motor complications and a lower risk of dyskinesia, particularly in early-onset PD [30,31,32]. However, long-term treatment with both L-dopa and dopamine agonists has been linked to the development of FoG in some cases [33,34].

In an observational study, FoG was distinctly rare in PD patients before L-dopa treatment, but after six years of using this form of treatment, its prevalence increased to 55% compared to a non L-dopa group [34,35,36]. The prevalence of FoG in patients who have never taken levodopa is lower than in patients who have been exposed to long-term use of LD, indicating a “levodopa paradox” [34]. FoG does not consistently respond to L-dopa and may occur in both the “on” and “off” time [37]. Patients have been observed to experience worsening “on” FoG when L-dopa dosage is increased [37]. However, the occurrence of FoG in levodopa-treated group may also be due to other factors such as disease duration, disease severity or other unexpected confounding variables [34].

The effectiveness of carbidopa-levodopa in controlling FoG appears to be patient specific as FoG persists in some individuals even in the ON state [30,31,32,33,38]. Patients in the ON state may experience a paradoxical increase in the number of FoG episodes [33]. Dopaminergic therapy may be most helpful for patients whose freezing occurs during OFF periods [39,40,41].

Levodopa-carbidopa intestinal gel (LCIG) therapy delivers continuous medication directly to the small intestine, providing stable dopamine exposure and reducing fluctuations associated with irregular tablet absorption. While LCIG can stabilize axial symptoms and FoG initially, long-term data suggest these improvements fade as the disease progresses. For example, after four years of LCIG, axial scores declined significantly, and 16% of patients became wheelchair-bound due to FoG and postural abnormalities despite continuous infusion therapy [41]. Methylphenidate, droxidopa, and amantadine have shown limited and inconsistent benefits and are not recommended as standard treatments, though they may still be considered on a case-by-case basis [42]. The limited effectiveness of medications in managing FoG is partly due to the involvement of non-dopaminergic systems. For example, dopa-unresponsive FoG has been linked to the degeneration of cholinergic areas, such as the pedunculopontine nucleus (PPN), nucleus basalis of Meynert (NBM), and posterior parietal cortex, suggesting a role for attention, postural control, and spatial awareness in FoG [43]. These findings further emphasize the need to explore alternative treatment strategies.

**Surgical Intervention:** DBS is a type of neuromodulation that provides constant stimulation to subcortical areas, helping restore motor function and reduce levodopa induced dyskinesia [33,44]. DBS electrodes are most often placed in the subthalamic nucleus (STN), followed by the globus pallidus interna (GPi), and the ventral intermediate nucleus (VIM) of the thalamus [2,28,33]. Based on the Unified Parkinson’s Disease Rating Scale (UPDRS III), DBS in the STN and GPi has shown approximately 40% improvement in motor scores during the off-medication state [44]. As a device-aided therapy, DBS is also associated with technical complications such as battery depletion, sudden cessation of stimulation, infections, and surgical risks that can cause severe rebound of PD symptoms [33].

## 3. Wearable Technologies in PD Management

Wearable technology has emerged as a potential therapeutic intervention for PD, especially for motor symptoms like FoG. Researchers have shifted to device-based measures that enable real-time monitoring and cueing when irregularities in motor output occur, as conventional treatments such as deep brain stimulation and dopamine replacement therapy often fail to effectively treat FoG [45]. 

Augmented reality prompting can project visual cues [25,46], while haptic vibrations [47] and metronomes associated with gait events [48] provide tactile clues. The closed loop cueing systems, which were responsive to the user’s gait, produced significantly better results for gait initiation, step length, and frequency of FoG incidents, according to a study that used tactile cues to compare an open-loop system with a closed-loop system [47]. Similarly, as compared to static cues or augmented light beams, AR smart glasses that used dynamic visual cues showed the best results for decreasing FoG episodes and improving gait rhythm [25]. Personalized, real-time cues that react to a person’s gait might be more effective than a continuous stimulus. A wearable device that used machine learning to accurately detect gait phases with 95% accuracy was able to activate cues only when necessary [48].

Machine learning algorithms, like Random Forest classifiers and Long Short-Term Memory (LSTM) networks are being deployed to predict FoG episodes [49,50], up to two seconds before they occur [51,52]. Wearable technology’s predictive capabilities will enable real-time interventions like automatic cueing or caregiver notifications so that actions can be performed in advance, thereby preventing falls or worse outcomes. For both clinical and home-based contexts, smartphone-based designs that allow for continuous monitoring of FoG have been developed in addition to these solutions. For instance, a study using a smartphone worn at the waist, without any extra hardware, detected FoG episodes with 91.5% accuracy, and tracked the episodes’ intensity and variations according to the time of day. All of this was done using a smartphone and with no specialized hardware [53]. 

Use of vibrotactile foot devices reduced the number of FoG episodes and percent time freezing by 33.1% and 32.6%, respectively [54]. Most of the studies on these wearable sensors have focused on better understanding the pathophysiology of FoG; however, there is increasing demand for these technologies to be used in the clinical characterization and management of FoG [55]. 

To assess fall risk in PD patients, Ayena et al. (2016) [56] developed a home system that combines smart insoles, an app, and a customized biomechanical model. To assess postural stability in terms of center of pressure (CoP) sway, neuromuscular delay, and sensory integration, tests for both static postural stability, such as the One-Leg Standing Test (OLST), and dynamic postural stability using a Tether-Release Test (TRT) were conducted. The OLST game encouraged regular balancing training in a fun, game-like way, and the Bluetooth insoles made it possible to send data remotely for additional research. People’s risk of falling was significantly influenced by the surface they stood on, and PD patients’ balance was worse than that of healthy individuals [56]. 

According to Pisano et al. (2024) [45], incorporating cerebellar transcranial direct current stimulation (ctDCS) into an augmented reality treadmill (C-Mill VR+) can help individuals with FoG, especially when their eyes are closed, by improving leg stability and step length. The sensor data from the treadmill showed slight but noticeable improvements in motor function, although traditional clinical tests did not reveal any significant changes. This group demonstrated the value of using advanced sensor-based tracking to identify motor performance changes that were not recorded by conventional evaluations. 

These non-invasive, affordable, and home-based FoG detection solutions offer a substitute for hospital-based monitoring and patient care personalization. Although C-Mill treadmills have shown benefits in research settings, their use is unlikely to be feasible in-home based contexts due to the high cost and the technical expertise required to operate the treadmill and associated tDCS systems. Wearable technology, particularly with AI or reactive cueing systems, has the potential to transform the way that people with PD manage their mobility and independence, despite certain drawbacks, such as the requirement for larger trials and environmental complexity as a potential confounding variable in cue effectiveness [57]. 

## 4. Current Approaches to Measure and Investigate FoG

Four different models have been proposed to explain the mechanism underlying FoG [5,58]. While the threshold model suggests that FoG occurs due to accumulation of motor deficits, the interference model argues that it is a result of disordered neuronal crosstalk between cognitive, limbic and motor input [5,13,58,59]. On the other hand, the cognitive model proposes that defects in conflict resolution lead to behavioral indecision and thereby result in freezing episodes [5,58]. Lastly, according to the decoupling model, a lack of synchronization between preparatory programming and intended motor response is suggested to cause a freezing episode [5,58]. Despite these proposed models, the mechanism of FoG is still poorly understood and needs more exploration. 

Two longitudinal studies found that non-FoG PD patients who have higher doses of dopaminergic medication and worse motor symptoms are more likely to develop FoG as the disease progresses [10,59]. Presence of motor fluctuations, as indicated by predictable and unpredictable wearing off of levodopa, alone in early stages of PD increases the risk of developing FoG by 3-fold [10]. Interestingly, in both transitional (participants who did not have FoG during the baseline but had developed it during the follow up) and continued freezers signs of cognitive dysfunction and sleep disturbances were observed [59]. While FoG is more common in advanced PD patients, 17% of freezers reported initially developing FoG within the first three years of diagnosis, which suggests that FoG can also occur in early stages of PD [60]. At 12 years post-diagnosis, 63% of the studied population (*N* = 232) experienced FoG [10]. 

FoG increases the risk of falling in PD patients [11,15,61,62], which increases injury risk and adds to both healthcare costs and caregiver burden. Contreras et al. [60] reported that 31% of freezers have reported falls due to a freezing episode whereas Sliva de Lima et al. (2017) found that 61% of falls in PD patients were related to FoG [63]. Due to FoG, patients also experience limited mobility, social interaction and independence which together results in overall reduction in their quality of life [12,17,19,64]. Furthermore, PD patients using ambulatory assistance devices reported a higher percentage of falls and an overall increase in economic burden by an average of $26,467 (year 2010 values) annually compared to non-PD controls [65]. 

### Clinical Assessment

Due to the sporadic nature of FoG, clinical assessment of FoG is difficult [18,61]. FoG is hypothesized to occur during automatic movement but in a clinical setting, walking becomes a goal directed behavior such that occurrence of FoG is rare and difficult to obtain [11,18]. Different techniques have been utilized to initiate FoG in clinical settings—these include dual task paradigm, turning, narrow passage, obstacle doorway, timed up and go test (TUG). Of these, 360 degree turning in place has been identified as the most effective technique to trigger a freezing episode [18,66,67,68]. However, occurrence of FoG during clinical evaluation is still sporadic and infrequent. Due to this reason, FoG has been assessed using subjective measures and relies on an observation of baseline gait and FoG, as opposed to sporadic FoG onset. The most used and reliable questionnaire is the Unified Parkinson’s Disease Rating Scale (UPDRS) item 14, which asks the patients to rate their frequency of falls that are related to freezing episodes [55,61,69]. FoG-Questionnaire (FoG-Q) and New FoG-Q (NFoG-Q) are relatively new questionnaires proposed to assess not only clinical aspects of FoG but also its impact on quality of life [61]. However, Shine et al. (2012) [69] found that NFoG-Q and FoG-Q scores did not correlate with FoG severity assessed by clinicians. 

The UPDRS employs three to four clinician “raters”, evaluating video recordings of ambulating PD patients to identify score gait and potential freezing episodes, thereby ascribing a an increasing score (0–4) of the FoG severity [55,61]. Normally, there is high agreement between raters, who are most likely from the same or collaborating institution [69], but when reliability between truly independent experienced raters was evaluated, the agreement rates were much lower [61]. This highlights that subjective assessment of FoG might not be an adequate and reliable source for FoG diagnosis and suggests the use of both objective and subjective measures for clinical assessment of FoG [5,18,61]. 

Inertial measurement units (IMUs), gyroscopes, and accelerometers are examples of technology that can continuously and longitudinally monitor movement-based gait parameters and ultimately help to monitor FoG in clinical setting [63,70]. One of the most promising objective measures for FoG is the vertical shank accelerometers [71]. The device works on the principle that FoG episode is mostly accompanied by trembling of legs, which produces high-frequency compared to locomotion or volitional standing [11,61,71]. The use of a vertical accelerometer was able to identify FoG with up to 89% accuracy when the device was calibrated for each subject [71]. While the accelerometer calculated the freezing index (FI) and quantified the number of FoG events, Morris et al. (2012) [61]. showed that percent time frozen recorded by the device was a better objective measure for FoG as it was strongly correlated with clinical mean rating of clinicians and was also found to have strong intra and inter rater agreement among truly independent raters. Furthermore, it has been demonstrated that three-day studies employing triaxial accelerometers worn in the natural setting are more effective at predicting future falls than standard clinical assessments like the Timed Up and Go (TUG) and UPDRS in individuals who have experienced falls within the last month or who have never experienced falls [70]. The capacity to identify small gait abnormalities in real-world scenarios supports the idea that gait quality—rather than just quantity—is a significant predictor of fall risk [70]. 

**Figure 2:**
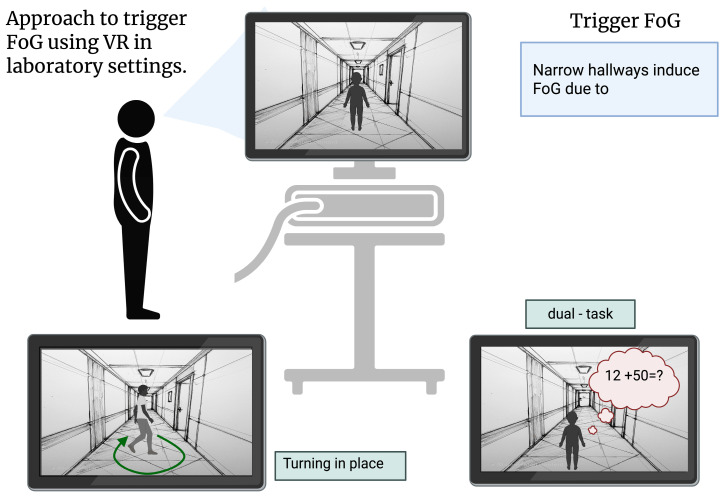
FoG can be triggered in laboratory settings using various environmental stressors, such as walking through a narrow hallway (**top**), increasing cognitive load (**bottom left**), such as dual tasking, and turning in place (**bottom right**).

To increase occurrence of FoG in a controlled clinical environment, recent studies have used VR technology to create FoG-inducing environments, such as doorways of different widths and dual-tasking cues to assess freezing in patients (Figure 2) [72,73,74,75]. Subjects who were exposed to such environments exhibited freezing and gait abnormality similar to those experienced in everyday life. Out of different cues, Gomez-Jordana et al. (2018) [73] found that 66% of trials using the VR environment with narrow doorways resulted in freezing episodes. However, if the patient was examined during “ON” state of dopaminergic medication, the number of FoG episodes in the narrow doorway condition reduced [75]. Use of VR to elicit FoG has also helped us better understand and characterize different gait disturbances observed in FoG such as increased foot latency, short and uneven steps, step variability and so on [72,73,75]. 

Additionally, there has been a growing need to identify characteristics of the “prefreezing phase” to prevent the occurrence of FoG [18,62]. Decrease in stride length and increase in cadence, together also known as festination, has been found to occur before FoG [64,76]. Coste et al. (2014) suggested a new approach called FoG criterion (FoG-C) for detection of FoG events, which includes continuous evaluation of gait parameters: cadence and stride length with the help of inertial sensors [62]. In a comparative analysis between FoG-C and FI, it was found that out of 26 main FoG events, FoG-C was able to identify 22 festination and FoG episodes whereas FI was only able to detect 17 [62]. The use of FoG-C can be extremely useful as the real time detection of festination can help us trigger cueing devices at the right time to prevent FoG episodes [18,62]. Further research is needed to evaluate the FoG-C approach, as it appears to be a promising avenue for managing FoG. 

## 5. Investigating the Usage of AR/VR for FoG Treatment

In laboratory settings, VR based gait tasks can be used to induce freezing episodes, which share similar neural substrates of FoG [77,78]. Yamagami et al. (2023) [78] found that VR environments, like doorway, hallway and crowd scenes, specially designed to provoke FoG can also cause increase in gait variability and festination, both of which are considered as the hallmarks of FoG. Aside from trigger FoG, most work with AR/VR focuses on reducing and/or reversing FoG through cueing strategies and training programs. With an emphasis on research conducted in the last ten years, we provide an overview of studies that have analyzed the effectiveness of various VR/AR technologies in reducing the debilitating symptoms of FoG. 

Altogether, we found that out of 17 articles examining AR/VR and FoG, 7 articles showed promising results in a reduction of FoG burden, whereas 8 showed no effect on FoG, and 2 articles showed mixed results. Herein, we summarize these data segregated by intervention type. 

### 5.1. AR 

Out of 7 studies (Table 1) focused on AR as FoG intervention, 2 studies showed positive effect of AR cueing on FoG [25,79], whereas 4 studies found no effect [12,80,81,82] and 1 study showed mixed results (Table 1 and Table 2) [83]. The most common AR technology (Table 2) used were head mounted devices which project visual cues onto the patient’s field of vision. Janssen et al. (2017) tested the effectiveness of AR cues to reduce FoG and gait impairments in comparison to conventional cues [12]. Two experimental cues: 3D augmented bars and 3D augmented staircase were projected by the smart glasses, whereas for control conditions, conventional 3D transverse bars on floor, an auditory metronome and no cueing were used [12]. Three walking tasks were performed by all participants while wearing the smart glass. AR cues did not change either the percent of time spent on FoG or the number of freezing episodes, potentially due to the heavy weight of the glasses used [12]. Additionally, the participants reported that the glass blocked their peripheral vision [12]. These variables may have masked the potential positive effect of AR cues. 

In a subsequent study, Janssen et al. (2020) [80] used an interventional single-patient case study design to investigate whether wearing the smart glass was hindering responsiveness to conventional cueing methods. Their participant was a 63 year old individual, who had experienced FoG for 16 years and had self-reported unresponsiveness to dopaminergic medication and DBS. On testing various cueing conditions, physical bars placed on the floor reduced the percent time frozen with or without worn smart glass [81], suggesting that wearing smart glasses did not mask the positive effect of conventional cues. However, like their previous study, AR cues did not improve or worsen FoG in the PD patient [81]. In another study, Janssen et al. (2020) [81] used the HoloLens to assess the influence of AR visual cues on FOG severity, axial kinematics and on scaling and timing of turning on 16 PD patients. The AR visual cue consisted of a small and large sphere. A “consuming” game was incorporated in the visual cue in order to trigger goal-directed movement, where participants were asked to move their body in order to “consume” the small sphere with the large sphere, while rotating 180 degrees in a small area as fast as they could [80]. Patients were then asked to wear HoloLens while turning in a small area with no cues, traditional metronome cues and AR cues. No effect of AR cues on percent frozen time (PFT), mean number and duration of FoG episode was observed [80]. While HoloLens was identified as a better alternative to all previous AR glass models, it was still unsuccessful to reduce FoG [80]. Additionally, its weight and aesthetics may prevent adoption. The study authors posit that the “consuming” game might have introduced a dual task effect rather than an integrated turning strategy, thereby masking the positive effect of AR visual cues [34]. Another important limitation of this study was the lack of habituation to the HoloLens and its cueing. Geerse et al. (2022) investigated this habituation confounder with a subsequent HoloLens study, by giving the participants 3 weeks to habituate to the device [79]. During the first session when patients were first introduced to HoloLens, they experienced longer FOG duration, longer frozen time and increased number of FoG episodes [79]. In contrast, during the third and last session, HoloLens significantly decreased PFT, total and average duration of FoG and additionally decreased the number of FoG episodes [79]. This work suggests that future work should incorporate a habituation phase in their study design. 

A study using the Google Glass (GG) hardware found no difference in the frequency and duration of FoG episodes among all cueing conditions [82]. This first model of GG generated three different cues: audiovisual cues (metronome), flashing light (LED) and optic flow. For the flashing light cue, participants were asked to take a step in accordance with a flashing light. For the optic flow cue, participants mirrored ambulation in response to a visual cue on a screen [82]. All three cueing conditions and a control condition (no cue) were tested in four different walking courses (wide turn course, narrow turn course, full turn course and doorway course) designed to elicit FoG. As the effect of cueing on FoG was dependent on the type of walking course, a reduction in the number of FoG episodes was only found during the complex 360 degree turns while using the metronome cue (*p* > 0.05) [82]. A key limitation in the study was the inconsistent presentation of FoG in the participants—with 4 out of 12 participants not experiencing a freezing episode and 2 participants only experiencing FoG once [82]. Additionally, the cues were displayed on the upper right corner of the glass, which required participants to focus attention on the corner while walking. Future studies should investigate whether the placement of AR cues impacts the effectiveness of AR intervention for FoG. 

In 2020, another GG glass model was introduced which consisted of the “Moving through Glass” software program. Lee et al. (2023) [83] designed an experimental setup to determine whether the latest GG prototype reversed FoG parameters. Participants were asked to walk under three different walking conditions designed to evoke FoG episodes, while using two different cueing programs of the software: “Walk with me”—with audiovisual cues and “Unfreeze me”—with audio cues in the form of songs. The “Walk with me” program improved scores on all walking tasks except for 180 degree turning tasks [83]. Comparatively, the “unfreeze me” program did not improve scores of walking tasks except for the dual task serial 7 straight walking task [83]. In both programs, participants observed a recorded video of a person walking with overlaid rhythmic audio cues. There was an increase in walking time [83]. However, this study did not calculate the number of freezing episodes, stride length, step variability or cadence but rather only focused on the walking time. 

More recently, an AR headset called Magic Leap 2 (ML2) facilitated the largest field of view compared to other previously tested headsets [12,25,79]. Baugher et al. (2025) [25] tested the AR cues projected by ML2 with traditional cue and no cue conditions in three walking conditions that were known to trigger freezing episodes. The AR cues were two concentric rings with different display conditions: constant cueing, eye controlled cueing, hand controlled cueing, and external observer-controlled cueing [25]. When the cue was controlled by the observer, participants spent least time frozen such that they were successful in overcoming freezing once the cue was provided [25]. While promising, observer controlled cueing poses a challenge in real-world scenarios, due to the need for continuous access to an observer with expertise in identifying FoG. On the other hand, with the use of constant cueing, participants reported lower rates of freezing suggesting that constant cueing can help reduce FOG incidence [25]. However, constant cueing can cause patients to get habituated to the cue [84], thereby masking any positive effects of cueing; therefore, a more dynamic cueing strategy is required. Finally, the AR cue preferred by the participants was the most successful in decreasing time spent frozen and providing a lower freeze rate in patients compared to other conditions [25]. 

In conclusion, there is inconsistent evidence supporting the effectiveness of AR intervention for rehabilitation of FoG. We base this conclusion on the 4 [12,80,81,82] out of 7 AR studies, which found no effect of AR cues in different FoG parameters. However, no strong conclusion can be drawn from these studies as there is a huge inconsistency in methodologies used to provoke and assess FoG as well as in the device used to provide AR cues. Most studies have highlighted that AR glasses posit as a major confounding variable due to their heavy weight, blocking of peripheral vision and placement of cues which can cause a dual task effect. Hence, development of light AR glasses with larger field of view is required to reduce the confounding effects reported in previous studies. Until then, incorporating a habituation period to the AR glasses might be more helpful in isolating the effect of AR cues as shown by Geerse et al. (2022) [79]. Additionally, participants should be stratified based on stage of disease and disease duration to identify whether the effectiveness of AR cues is dependent on the progression of FoG [79].

Due to the limited number of studies and the large amount of heterogeneity present in them, making a generalized conclusion about the effectiveness of AR cues on FoG parameters is difficult. Therefore, a more consistent and standard methodology is required to fully evaluate the rehabilitative potential of AR technology for FoG. 

### 5.2. VR 

Of the studies reviewed (Table 3), five were repeated training interventions either via treadmill based VR, action observation, or cognitive-motor training [43,75,76,78]. Through repeated training sessions, these training programs sought to improve dual-tasking skills, improve gait, and/or lower participants’ risk of falling. 

VR-based multimodal training, along with treadmill training (TT+VR) had beneficial effects on fall risk compared to standard treadmill walking (TT) alone in fall-prone elderly adults, some of whom had PD [85]. Bekkers et al. (2020) [86] compared the effect of two training arms (TT+VR and TT alone) on PD patients with and without FoG. While all training arms and PD groups showed improved postural stability and general mobility at post-intervention, these improvements were not retained after training at 6 months follow-up. Both groups receiving TT+VR training reported better mobility and a reduction in the number of falls [86]. However, FoG severity increased in both training arms at 6 months follow-up. The VR+TT consisted of motor training and cognitive training; however, these did not alleviate FoG in patients at 6 months follow-up [86]. 

Killane et al. (2015) examined the effect of motor and cognitive training on dual-task performance in PD patients with and without FoG [87]. The training task consisted of a VR maze in which navigation was controlled by stepping on a balance board and pressing buttons on a remote to respond to a screen-administered Stroop test [87]. After receiving eight sessions of training, both groups were tested in three different tasks: single motor task, a single cognitive task, and a dual motor-cognitive task. The training program decreased the number of FoG episodes in dual tasks; however, it was not statistically significant (*p* = 0.10), but there was no change in the number of FoG episodes during single tasks (*p* = 0.57) [87]. 

Despite the reported decrease in freezing episode during dual task, it can be difficult for physicians operating in a clinical context to introduce this training into everyday practice. According to Domingos et al. (2022), many therapists that assist patients in executing their dual-task training reported encountering challenges such as limited time for therapy fulfillment, safety concerns, and a need to comprehend how to adjust the difficulty of the task based on patient’s stage of the disease [88]. Furthermore, patients with advanced PD experiencing cognitive impairment create even greater challenges for clinicians to effectively provide dual-task training [88]. Executing dual task VR training should be considered carefully as patients in advanced stages of PD groups would probably be more prone to freezing episodes and falls if they tried to complete their walking task while doing another cognitive task because walking already requires a significant amount of their mental abilities [88]. 

Goh et al. (2021) proposed a new VR self-modeling intervention [89]. This observational learning model reinforces a patient’s reversal of FoG by showing the patient pre-recorded videos of themselves successfully overcoming FoG. In this experimental design, 10 participants identified a home-based FoG-eliciting activity and received a personalized strategy to overcome the FoG episode with the help of a trained physiotherapist. Then, an immersive and personalized VR video was engineered showing the successful performance of the previous FoG-eliciting task. As a part of the training, participants were asked to watch the video at least twice a day and to practice the intervention strategy for 10 min. After 6 weeks of training, minimal averaged changes were observed in FoG, mobility, and anxiety; however, substantial variability among participants was observed, with one participant reporting a reduction in PFT by 94% to 84% and another reporting an increase in PFT by 22% to 27% [89]. Regardless, all participants qualitatively reported greater confidence in resolving FoG-triggering activities during post-intervention feedback. They also reported lowered anxiety, as measured by Parkinson Anxiety Scale (PAS), during FoG onset, potentially due to knowledge of FoG reversal strategies [89]. Considering the limited sample size and variability, retention (9/10) and adherence (video watching = 84% ± 49% and physical exercise = 100% ± 56%), a larger-scale longitudinal study is required to fully understand the effect of VR self-modeling intervention and FoG parameters. 

Another effective technology used to reduce FoG episodes is the use of VR light cueing lasers. A recent study by Barthel et al. (2018) found that the use of laser shoes can improve PFT by 56.5% during the OFF state (*p* = 0.04) and by 51.4% during the ON state (*p* = 0.075) [90]. The laser cue is projected orthogonally to the leg that is about to move. It is a step by step cueing device which is activated during the heel strike and is turned off once the body weight is removed from the heel. When laser cueing was used, FoG episodes also reduced by 45.9% during the OFF state and by 37.7% during the ON state [90]. The use of laser shoes can be especially effective in the PD population having major cognitive impairments as the benefit of the laser shoes has been found to persist regardless of the patient’s frontal cognitive abilities.

Across the studies, VR was generally associated with improvements in gait-related metrics such as increased step length, reduced variability, and improved dual-tasking performance [85,87]. Lei Ma et al. (2025) [91] used immersive VR to compare real-world and virtual walking across several tasks, and determined that although FoG episodes could be reliably triggered in both settings, the frequency of freezing was comparable between VR and in-person (real world walking), specifically for walking around obstacles (mean difference of 4.18 s, *p* = 0.03). This suggests that immersive VR can accurately mirror realworld triggers for FoG development in a controlled environment, possibly because patients have to concentrate more in VR due to unfamiliar visuals, limited body awareness, and the extra effort needed to process the virtual environment. However, there were conflicting results about VR’s ability to lower FoG episodes [91].

Most studies on the use of VR for reducing FoG have shown inconsistent results. Of the 6 studies, 5 showed some improvement in gait-related outcomes (specifically, balance, and dual-task performance) [45,86,87,89], 1 showed a non statistically significant decrease in number of freezing episodes and only 1 study with VR laser shoes reported a statistically significant decrease in episodes of FoG [91]. These improvements were temporary and differed greatly between participants [86]. However, differences in VR systems, task difficulty, training duration, and inconsistent outcome measures make conclusory comparisons difficult [85,86]. 

Overall, research suggests that rather than directly addressing the symptoms of freezing gait, VR mostly improves walking and balance and lowers the risk of falls [4,86,87]. However, it has not been shown to lessen the frequency of freezing episodes in people [5]. Future studies should use more consistent methods, follow patients for longer periods, and clearly identify which patients are most likely to benefit from VR training. This conclusion should be interpreted carefully, though. It is only based on the research that we have already reviewed. This does not exclude VR from being more useful in the future as research designs and technology advance. 

**Table 3:** Studies investigating the use of VR to address, manage, or treat FoG. Abbreviations: VR—Virtual Reality, MDS-UPDRS III—Movement Disorder Society-Sponsored Unified Parkinson’s Disease Rating Scale III, UPDRS III—Unified Parkinson’s Disease Rating Scale III, FoG—Freezing of Gait.

Author, Year	Type of VR	Treatment Condition	Population Characteristics	FoG ON/OFF	Outcome
Barthel, 2018 [90]	Laser shoes	Patients performed 5 different gait protocols with laser shoes turned on and turned off	*n* = 19; Age: 68.68 ± 11.15 years, Disease Duration: 11.21 ± 6.68, MDS-UPDRS III: OFF—36.21 ± 12.80, ON—45.68 ± 10.30	Some ON, some OFF	FoG episodes reduced by 45.9% during the OFF state and by 37.7% during the ON state
Bekkers, 2020 [86]	Treadmill	•Exercised 3 times/week x 6 weeks •Training arms: traditional treadmill (TT) + VR and TT alone •TT + VR group trained on complex interactive obstacle negotiation and cognitive load training with visual and auditory feedback •Balance, falls, FoG measures were taken pre and post intervention and at 6 months follow up	**Two PD groups** (1) FoG + (*n* = 77; Age: 70.57 ± 6.04 years, Disease Duration: 10.43 ± 6.7, UPDRS III: 31.83 ± 13.2) (2) FoG - (*n* = 44; Age: 71.66 ± 6.3 years, Disease Duration: 7.25 ± 5.1, UPDRS III: 26.11 ± 12.2) **Two training arms** (1) TT+VR (*n* = 62; Age: 71.66 ± 6.3 years, Disease Duration: 9.05 ± 5.5, UPDRS III: 30.11 ± 13.1) (2) TT alone (*n* = 59; Age: 70.86 ± 6.0 years, Disease Duration: 9.55 ± 7.2, UPDRS III: 29.37 ± 13.2)	ON	**TT +VR group at 6 months** reduction in the number of falls in both FoG+ and FoG. **Both training arms** FoG severity increased at 6 months.
Goh, 2021 [89]	Video	•Participants watched a video of themselves overcoming FoG 2x a day •Intervention was followed for 5 days/week for 6 weeks	*n* = 10; Age: 70.6 ± 7.7 years, Disease duration: 13.3 ± 5.2, UPDRS III: 37.3 ± 13.3)	ON	No effect, but variation in response among participants reported
Killane, 2015 [87]	Virtual maze	•Patients received eight session of motor (VR maze) and cognitive training (Stroop test) •Measurements were taken during three different tasks: single motor task, single cognitive task and dual motor-cognitive task	(1) FoG + (*n* = 13; Age: 64.2 ± 2.4 years, UPDRS III: 31.8 ± 2.8) (2) FoG - (*n* = 7; Age: 64.0 ± 1.6 years, UPDRS III: 22.3 ± 3.2)	N/A	Number of FoG episode decreased in dualtask (*p* = 0.10) but remained the same in singletask (*p* = 0.57)
Pisano, 2024 [45]	VR + stimulation	•Both groups received C-Mill (cerebellar neuromodulation) training •Training session involved application of tDCS or sham tDCS combined with a gait rehabilitation program via C-Mill •Motor cognitive training focused on enhancing dual-task performance while walking	Cerebellar Group (*n* = 9; Age: 71 ± 8.6 years Sham Group (*n* = 8; Age: 65.3 ± 8.5 years All groups experienced FoG	ON	Reduced freezingrelated gait impairments

## 6. Limitation and Future Directions

To manage symptoms of PD and improve quality of life, PD patients need long-term rehabilitation; however a constant challenge is the lack of adherence and retention to the rehabilitation program as they can get monotonous and boring over time. The use of VR/AR technology addresses this problem by using gamified exercise goals and creating challenging environments, which provides motivation and enjoyment to the patients [92,93]. Regardless, the long term effectiveness, user compliance and adherence of such rehabilitative techniques still remains unclear. Additionally, concerns about device accuracy, affordability, and sustainability remain. Implementation of these modern technologies in an elderly population possesses another challenge especially in advanced PD patients experiencing cognitive impairments [94]. The acceptance and utility of AR/VR in elderly population should be considered while designing the rehabilitation program. Despite an overall excited and engaged user experience with AR/VR, participants have reported that the device is often too heavy and that it interferes with their range of vision, thereby causing more complications in gait parameters [12,80]. New AR/VR technologies are trying to address this issue by ensuring that the device is lightweight, convenient and comfortable to use for an extended period of time, especially in FoG treatment and management, where patients need constant cueing. Due to constant use, it is essential for the device to have extended battery life such that it can be used continuously [93]. However, little to no attention has been given to issues regarding battery usage and concerns about sustainability as most of the rehabilitative technologies are in the trial phase and focus solely on effectiveness in treating symptoms. 

While the use of AR/VR is a new promising neurorehabilitation field, its integration with existing healthcare infrastructure requires a very lengthy and costly approval process. These new devices will have to go through strict approval procedures and demonstrate their safety and effectiveness before being approved to be used commercially [93]. The occurrence of an adverse effect during VR-based rehabilitation training is rarely reported; however, cases of visual hallucinations, strained eye and motion sickness have been reported in the existing literature [78,92]. Future studies should also address whether gait stability is different in patients while using AR vs VR headsets and its potential side effects on other gait parameters. Additionally, enrollment strategies should stratify participants in accordance with disease stage and disease duration to identify whether the effectiveness of VR cues is dependent on the progression of FoG [86]. VR/AR treatment can be very useful, specially in places with limited healthcare as the VR/AR device can record data (gait patterns), which can be later assessed by professionals remotely and they can provide personalized treatment and rehabilitation plans [92,95]. However, AR/VR devices can only be widely incorporated into clinical practice, once they become more affordable and widely distributed. To further optimize wearable technology, AR/VR technology should provide real time gait analysis and detection of festination, which can help predict FoG and therefore, provide cueing at the right time to prevent a freezing episode. Continuous monitoring of gait parameters can help with proactive symptom management and enhance functional independence in PD patients. 

## Figures and Tables

**Table 1:** Studies investigating the use of AR to address, manage, or treat FoG. Abbreviations: AR—Augmented Reality, MDS-UPDRS III—Movement Disorder Society-Sponsored Unified Parkinson’s Disease Rating Scale III, NFOG-Q—New Freezing of Gait Questionnaire, UPDRS—Unified Parkinson’s Disease Rating Scale, FoG—Freezing of Gait, DA—Dopamine Agonist, DBS—Deep Brain Stimulation.

Author, Year	Type of AR	Treatment Condition	Population Characteristics	FoG ON/OFF	Outcome
Baugher, 2025 [25]	Magic Leap 2	6 different cues: 4 AR cues (Constant AR cue, Eye controlled cueing, Hand controlled AR, Observer controlled AR), 1 traditional cue and no cue 3 Difficulty levels related to cognitive load Participants walked in a holographic hallway	*n* = 36; Age: 68.0 ± 9.1 years, Disease Duration: 7.7 (range: 0.3–20.3), MDS-UPDRS III: 41.8 ± 16.3	Some ON, some OFF	Observer-controlled cue condition reduced freezing time
Geerse, 2022 [79]	HoloLens	**Three sessions, scheduled 1 week apart** •Session 1 (home environment): Device habituation •Session 2 (lab setting): Personalization •Session 3 (home environment): Assessment	*n* = 25; Age: 67.0 (range: 55–76), Disease Duration: 15.4 (range: 7–31), NFOG-Q: 18.8 (range: 11–26), UPDRS III: 40 (range: 15–59)	ON	**Session 1**: Walking without habituation increased FoG **Session 1 vs. 3:** Habituation to HoloLens reduced FoG
Janssen, 2017 [12]	Smart Glass	•5 different cues; 2 experimental cues (3D Augmented bars and 3D Augmented staircase) and 3 control cues (conventional 3D transverse bars on the floor, auditory cueing via a metronome, and no cueing) •Three walking courses: walking straight, no additional task, stop and start, turning	*n* = 25; Age: 72 (Q1: 65–Q3: 79), Disease Duration: 11 (Q1: 3–Q3: 20), Years since FOG: 2 (Q1 −0.25–Q3: 12), UPDRS III: 34 (Q1: 10–Q3: 61)	End of dose Period	Improvement in‘conventional bars’ condition.
Janssen, 2020 [80]	Smart Glass	•With or Without glasses •Patient traverse a doorway four times under 7 conditions of cueing.	“*n* = 1; Age: 63, Disease duration: 17 years, self-reported FoG, unresponsive to DA and DBS”	OFF	No effect
Janssen, 2020 [81]	HoloLens	•Participants asked to “consume” a small sphere with a large sphere via movement •Three groups; experimental group with AR cues, control group with conventional metronome and control group with no cue	*n* =16; Age: 69 (IQR-13), Disease Duration: 10 (IQR-9), UPDRS part III: 38 (IQR- 17), NFOGQ: 18 (IQR-7)	End of dose period	No effect
Lee, 2023 [83]	Google Glass	•“Walk with me” program provided visual and auditory cues, whereas “Unfreeze me” program provided various musical songs and marching movements as cues •Control—No glasses, Experimental—Use of glasses; •180-degree turn after walking the 25 feet, dual tasks of walking in a straight line and verbalizing serial 7 subtractions from 100, and walking in a narrow passage	*n* = 9 (8 idiopathic PD); Age: 70.7 ± 10.0; Disease duration: 7.4 ± 5.5)	N/A	•“WalkWith Me” improved all walking tasks except turning. •“Unfreeze Me” worsened all walking tasks except dual task serial 7.
Zhao, 2016 [82]	Google Glass	•Single-session walking test with visual cue (blinking) and auditory (rhythmic tone) cues.	*n* = 12; Age: 66.8 ± 6.8 years, Disease Duration: 12.6 ± 6.7, UPDRS III: 35.2 ± 10.6)	End of dose period	No effect

**Table 2:** Commonly used AR hardware to study FoG include commercial off the shelf (COTS) platforms such as Google glass, Hololens, Smart glass and Magic leap 2.

AR Glass	Company	Duration of FoG	Frequency of FoG	Time Frozen	Other Gait Parameters
Google glass	Google X	No effect	No effect	N/A	All cueing reduced cadence and stride length variability
		N/A	N/A	N/A	•“Walk With Me” program improved all walking task scores except turning •““Unfreeze Me” program worsened all walking tasks except dual task serial 7 straight walking”
Smart glass	Cinoptics	No effect	No effect	No effect	N/A
		No effect	No effect	No effect	N/A
HoloLens	Microsoft	No effect	No effect	No effect	•reduce peak angular velocity •reduced step height •increased step height coefficient of variation
		Decreased	Decreased	Decreased	N/A
Magic Leap 2	Magic Leap	N/A	Decreased (only for constant AR cue and patient preferred cueing)	Decreased (only for observer controlled cueing and patient preferred cueing)	N/A

## Data Availability

No new data were created or analyzed in this study.
